# Supplementary material for: A CAF40-binding motif facilitates recruitment of the CCR4-NOT complex to mRNAs targeted by Drosophila Roquin
Source: Nat Commun. 2017 Feb 6;8:14307. doi: 10.1038/ncomms14307 (PMC5303829; doi:10.1038/ncomms14307)
Supplement: Supplementary Information — Supplementary Figures, Supplementary Tables and Supplementary References [file ncomms14307-s1.pdf]

Supplementary Figure 1

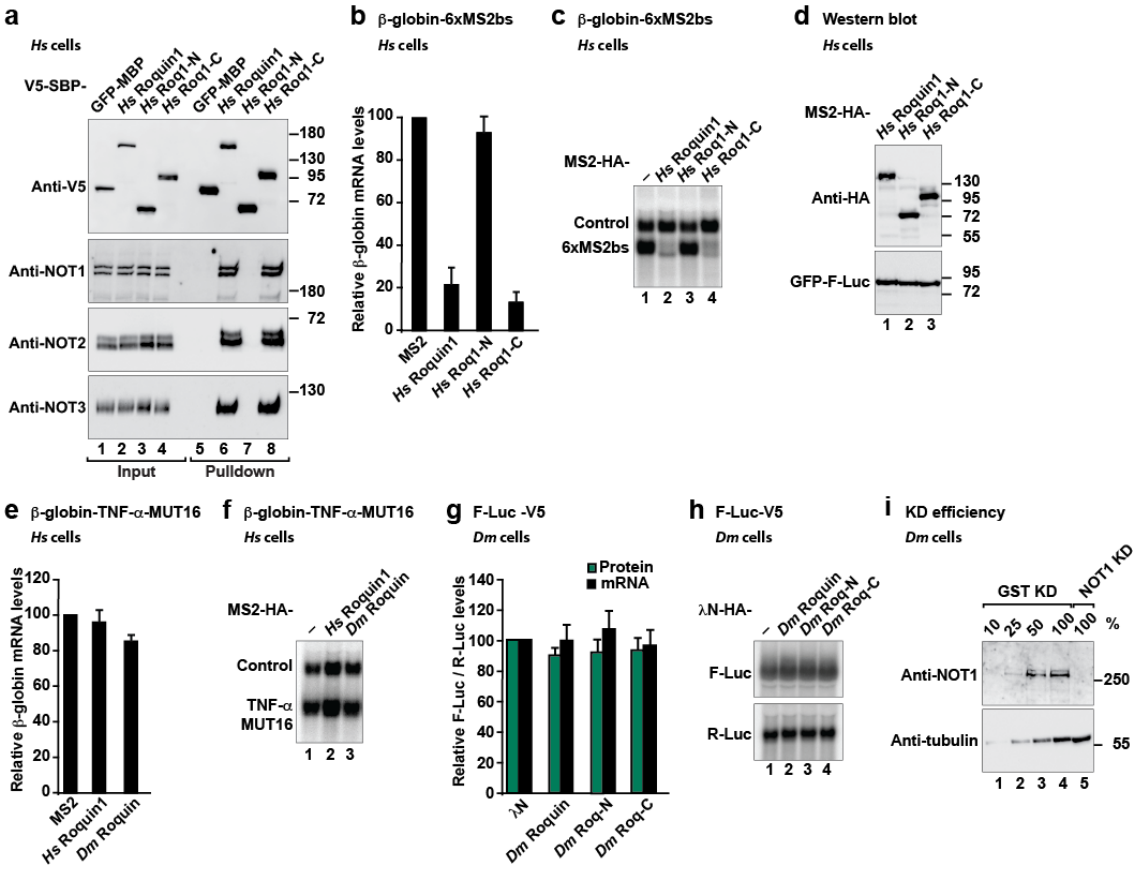

Supplementary Figure 1 | The C-terminal regions of Roquin proteins recruit the CCR4-NOT complex and trigger degradation of bound mRNA. (**a**) SBP pulldown assays in HEK293T cell lysates showing the interaction of V5-SBP-tagged *Hs* Roquin1 (full-length or the indicated fragments) with endogenous NOT1, NOT2 and NOT3. Input and immunoprecipitates were analyzed as described in Fig. 1b–d. (**b,c**) Tethering assays using the β-globin-6xMS2bs reporter and MS2-HA-tagged *Hs* Roquin1 (full-length or the indicated fragments) in human HEK293T cells. A plasmid expressing a β-globin mRNA reporter lacking MS2-binding sites (Control) served as a transfection control. The β-globin-6xMS2bs mRNA levels were normalized to those of the control mRNA and set to 100% in the presence of MS2-HA. The mean values ± s.d. from three independent experiments are shown in panel (b). Panel (c) shows a representative

northern blot. (**d**) Western blot showing the equivalent expression of the MS2-HA-tagged proteins used in the tethering assay shown in panels (b,c). (**e,f**) Effect of *Hs* Roquin1 and *Dm* Roquin on the expression of the β-globin-TNFα-MUT16 mRNA reporter carrying a mutated CDE (MUT16; ref. 10) analyzed as described in Fig. 1k,l. (**g,h**) A tethering assay using the F-Luc-V5 (lacking the 5BoxB sites) reporter in *Dm* S2 cells co-expressing λN-HA-*Dm* Roquin (full-length or the indicated fragments) was performed as described in Fig. 3a,b. (g) Mean values ± s.d. from three independent experiments. (h) Northern blot of representative RNA samples. (**i**) Western blot analysis of S2 cells depleted of NOT1 corresponding to the experiment shown in Fig. 3h,i. Dilutions of control cell lysates were loaded in lanes (1–4) to estimate the efficacy of the depletion. Tubulin served as a loading control. KD: knockdown.

Supplementary Figure 2

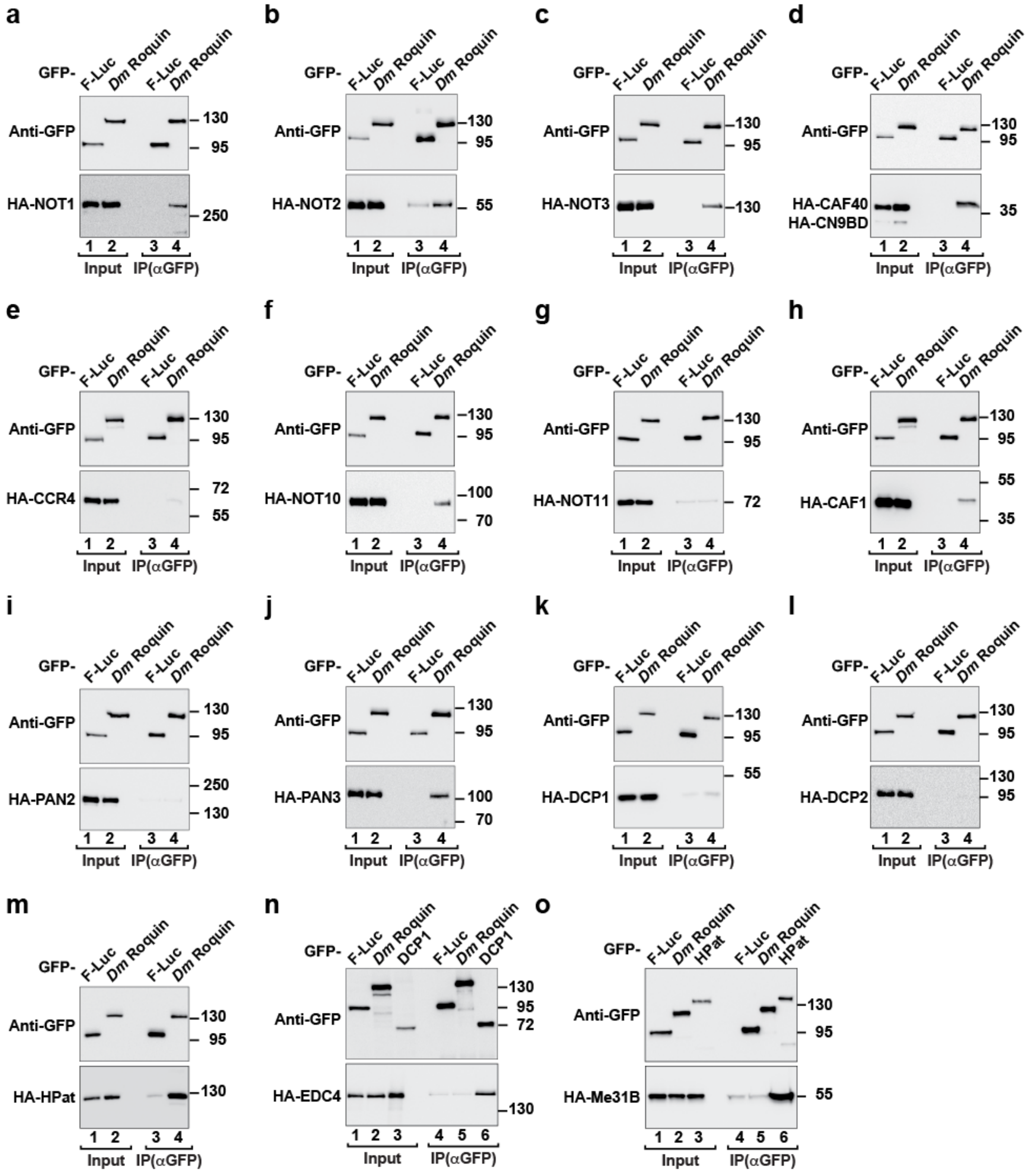

Supplementary Figure 2 | *Dm* Roquin interacts with subunits of the deadenylase and decapping complexes. (**a–o**) Co-immunoprecipitation assays showing the interaction of GFP-tagged *Dm* Roquin with the indicated HA-tagged proteins in *Dm* S2 cell lysates treated with RNase A. In panels (n) and (o), GFP-tagged DCP1 and HPat,

respectively, served as positive controls. In all panels, F-Luc-GFP served as a negative control. Protein size markers (kDa) are shown on the right in each panel.

Supplementary Figure 3

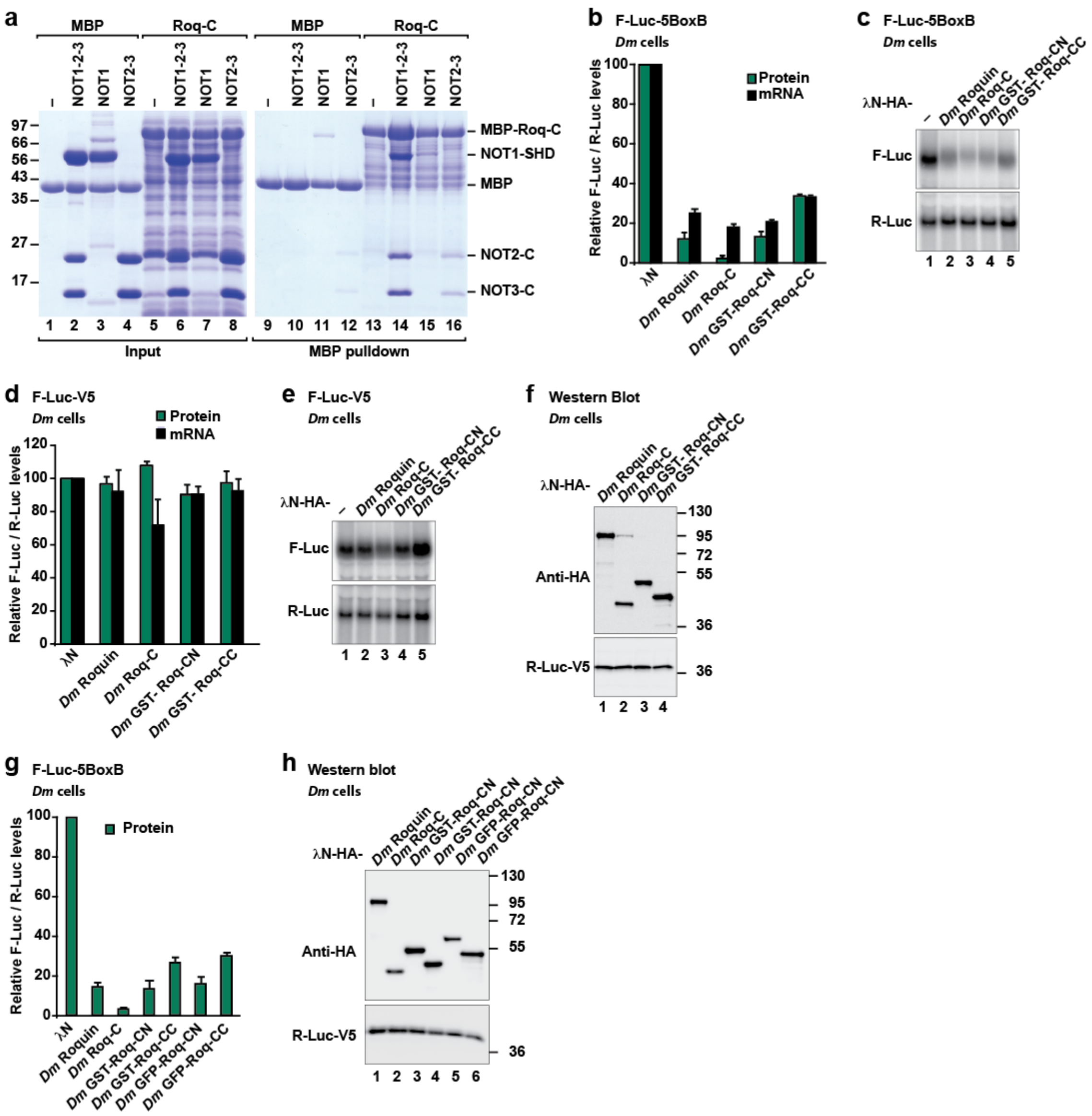

Supplementary Figure 3 | The *Dm* Roquin C-terminal region binds to CAF40 and to the NOT-module. (**a**) *In vitro* MBP pulldown assay showing the interaction of the MBP-tagged *Dm* Roq-C with the assembled NOT module (NOT1-2-3), the isolated NOT1-SHD (NOT1) and the NOT2-NOT3 dimers (NOT2-3). MBP served as a negative

control. (**b–e**) Tethering assays using the F-Luc-5BoxB or the F-Luc-V5 (lacking the 5BoxB sites) reporters and λN-HA-tagged *Dm* Roquin (full-length or the indicated fragments) were performed in *Dm* S2 cells as described in Fig. 3a,b. A plasmid expressing R-Luc served as a transfection control. F-Luc activity and mRNA levels were normalized to those of the R-Luc transfection control and set to 100% in cells expressing the λN-HA peptide. Panels (b,d) show mean values ± s.d. of the normalized F-Luc activities and mRNA levels from three independent experiments. Panels (c,e) show northern blots of representative RNA samples. (**f**) Western blot analysis showing the equivalent expression of the λN-HA-tagged proteins used in panels (b–e). (**g**) A tethering assay using the F-Luc-5BoxB reporter and λN-HA-tagged *Dm* Roquin (full-length or the indicated fragments) was performed in *Dm* S2 cells as described in Fig. 3a,b. The *Dm* Roq-CC and Roq-CN contained in addition either a GST or a GFP tag, with the purpose of visualization. A plasmid expressing R-Luc served as the transfection control. F-Luc activity was normalized to that of the R-Luc transfection control and set to 100% in cells expressing the λN-HA peptide. The panel shows mean values ± s.d. of the normalized F-Luc activities from three independent experiments. The GST or GFP tags do not interfere with the activity of the fragments. (**h**) Western blot analysis showing the equivalent expression of the λN-HA-tagged proteins used in the tethering assays shown in panel (g).

Error bars represent s. d. from three independent experiments.

Supplementary Figure 4

```
D.melanogaster   501 HYMGSP------RGYLDPS-----LGLSPGG-GLPP---------SHHSPITR------------LIV--  535
D.yakuba         500 HYMGSP------RGYLDPS-----LGLSPGG-GLPPQ-QSPLLHPSHHSPITR------------LVV--  542
D.ananassae      503 HYMASP------RSYIEPS-----LGLSPGG-GIPPPGHPPLLHPSHHTPMNR------------LMV--  546
D.pseudoobscura  486 HYMASP------RGYLDPNANLPGLGLSPGA-THPPQ--PPQLL--HHPSIARQ--------MNGLMV--  534
D.mojavensis     488 RYMQPPPPAPPARGFLDPN-----CNMPPAAHNLSPGAVHPQMLSAHHSPMGRQQQQQPHTALNGLLVPP  552
D.virilis        504 RYMQPP--AQPTRGFLEPN-----CSIPPAAHNLPPSGVHPQLLRAHHSPIGRQQQQQ-HAALNGLLVPP  565

D.melanogaster   536 -PS--RYDSRFSGFGGGTP----RIPSPREYQAN-PVA----PTQRN-ANPPNFS------------VNS  580
D.yakuba         543 -PS--RYDPRFSGFGVGTP----RNPSPREYQAN-P-A----PAQRH-ANPPNFS------------VNS  586
D.ananassae      547 -PG--RYDPRFAGYGVGQP----RNPSPREYQNN-PGAPPPPPPQRN-ANPPNFS------------VNS  595
D.pseudoobscura  535 -PG--RFDPRFN-YGPGQPIAQSRNPSPREYQAS-PGP----PQQRNLSNPPSFN------------VNS  583
D.mojavensis     553 PPPPLGYEQRFN-FAP--PP---RNAAR--------------------INPPNYNSNSNNSSNSNHSNNN  596
D.virilis        566 -PG--RYEQRFN-YGP--APAPSRNNATREYPGNSPTQ----PPQ---RNPPNYN---NISNNNNNNNNN  620

D.melanogaster   581 NLH---KGY--MLPASGGDVFH-----------------------LA------NPWEQ-AYLA--QQQH-  612
D.yakuba         587 NLH---KGY--MLPGGGGDVFH-----------------------LG------NPWEQ-AYLAQQQQQN-  620
D.ananassae      596 SLH---KGY--MLP-GGDSLFH-----------------------LG----PTHPWEH-GYPHPHQQHP-  630
D.pseudoobscura  584 NLH---KGY--MLP---ADVFHPTAYGYGS------------EEKLG------HPWEQHPYQQQQHQQQQ  627
D.mojavensis     597 NLHNH-KSFCSMLP---ADVYHAPYFGNEA----KGNVEM--SQKLG----GNNPWEH-PYM--------  643
D.virilis        621 NIHNHNKSFCSMLP---TDVYHSPYFGNEATGNSKGNAELPAATKLGHPAASGNPWEH-TYM--------  678

D.melanogaster   613 PPQHPQQQQP---PSSK--PNPSRPLS-ILPATADTSFFEKKPP-NSVSIDLDRVPEVN-----------  664
D.yakuba         621 PQPHPQQQQS---PSSK--PNPSRPLS-ILAATADTSFYEKKPP-NSVNIDLDRVPEVD-----------  672
D.ananassae      631 PQQQQQQQQPPQLPSSKPNPNPSRPLS-ILAATADTSFFEKKPP-NSVNLDVD-ADVTD-----------  686
D.pseudoobscura  628 QPQQQQQQPP---PSSK--PNPSRPLS-ILAATADTSFYEKKPP-NNVSIDMDMAKPLASLVASGSGAGE  690
D.mojavensis     644 -PTLVPPPPP---PSSK---NPSRVMSTISHATADTSFYEKKPP-STVNIEPLAEGAVN-----------  694
D.virilis        679 -PQALATLPP---PSSK---YPSRVMSTILPATADTSFYEKKPPSNNVNIDLDVESAAH-----------  730

D.melanogaster   665 -----VDAVPLFRRSN-----NNHNNNNSNSHN---NNNNNHGSSLLFWNNTGKDSANFVRSDSILDDDA  721
D.yakuba         673 -----VDAVNLFRRSN--------NNNNTSSH-----NNNNHGSSLLFWNNTGKESANFVRSDSILDDDA  724
D.ananassae      687 -----VDPLSRFRRSNINNS-SNNNNNNNSSSHNNNHNNNNNHGSSLLFWNNSSKESANFVRSDSILDDDA  750
D.pseudoobscura  691  AGDVLDALPLFRRFYI----QSHNHNHSHSHG---HSQGQNNSSLLF----------FERSDSILNDDG  743
D.mojavensis     695 -----SESMPLFRP---SNTGSTNNSNNNCSTN---NNN-----SLIFWNNSNKDSANFVRSDSILDDDA  748
D.virilis        731 -----AESMPLFRPNNNNNNSSSGNNNNSSSSN---NNNNNNNISLIFWNNSNKDSANFVRSDSILDDDA  792

D.melanogaster   722 STFDVPTGSSMLSIYGPICPKSSTTGNWNNF--------DLGY-GGFKSDRNDSFNANKQQQPMWGRKPQ  782
D.yakuba         725 STFDVPTGSSMLSIYGPICPKSSTTGNWNNF--------DLGY-GGYKSDRNDNFNANKQQQPVWGRKPQ  785
D.ananassae      751 TTFDMPTGSSMHSPYGPICPKSSTTNSWNNC--------DLGY-GGFKSDRNDNYNANKLQQPIWGRKPQ  811
D.pseudoobscura  744 STFDMPTGSSMHSRYGPICPKNSAASNWNNWIMENDTSSDLGY-AGYKADRNDNFNSHKSSQ-MWGRKPQ  811
D.mojavensis     749 STFDVPSGSSINSRYGPICPKRNVTNNWNNW---------LPK-PGYKPEHNDNTNANKQQ----DRKPV  804
D.virilis        793 STSDVPTGSSMHSRYGPICPKRS-TTNWHNW--------PMEHDPNNKNDSSEPAGSHNERLKQLDGKPM  853

D.melanogaster   783 LMSEDSFEGGIDSGMMLQLEKNLVDIVD----PDDSGIKVD  819
D.yakuba         786 LMSEDSFEGGIDSGMMLQLEKNLVDIVD----PDDSGIKVD  822
D.ananassae      812 LMSEDSFEGGIDSGMMLQLEKNLVDIVD----PDDSGIKVD  848
D.pseudoobscura  812 LLSEDSFEGGIDSGMMLQLEKNLVDIVD----PDDSGIKVD  848
D.mojavensis     805 LMSEDSVEGGIDSGMMSELEKKLVDIVEVWSNPDDSGIKLD  845
D.virilis        854 LLSEDSLEGGIDSGMMSELEKNLVDIVEVWSNPDDSGIKLD  894
```

CN ←|→ CC

NOT module binding motif (NBM)

CAF40 binding motif (CBM)

Supplementary Figure 4 | Sequence alignment of the *Drosophila* Roquin C-terminal regions. The CBM helix as determined from the crystal structure is indicated in red above the sequences. The secondary structure elements as predicted by PSIPRED (http://bioinf.cs.ucl.ac.uk/psipred/) are indicated in black. Residues conserved in all of the aligned sequences are shown with a dark green background, and residues with >70% similarity are highlighted in light green; conservation scores were calculated using the SCORECONS webserver[1]. The sequences required for binding to the NOT module (NBM) and the CAF40-binding motif (CBM) are indicated. Residues in the CBM that directly contact CAF40 are marked by brown diamonds. Residues that were mutated in this study are indicated by red asterisks. The boundary between the CN and CC fragments is indicated below the alignment.

Supplementary Figure 5

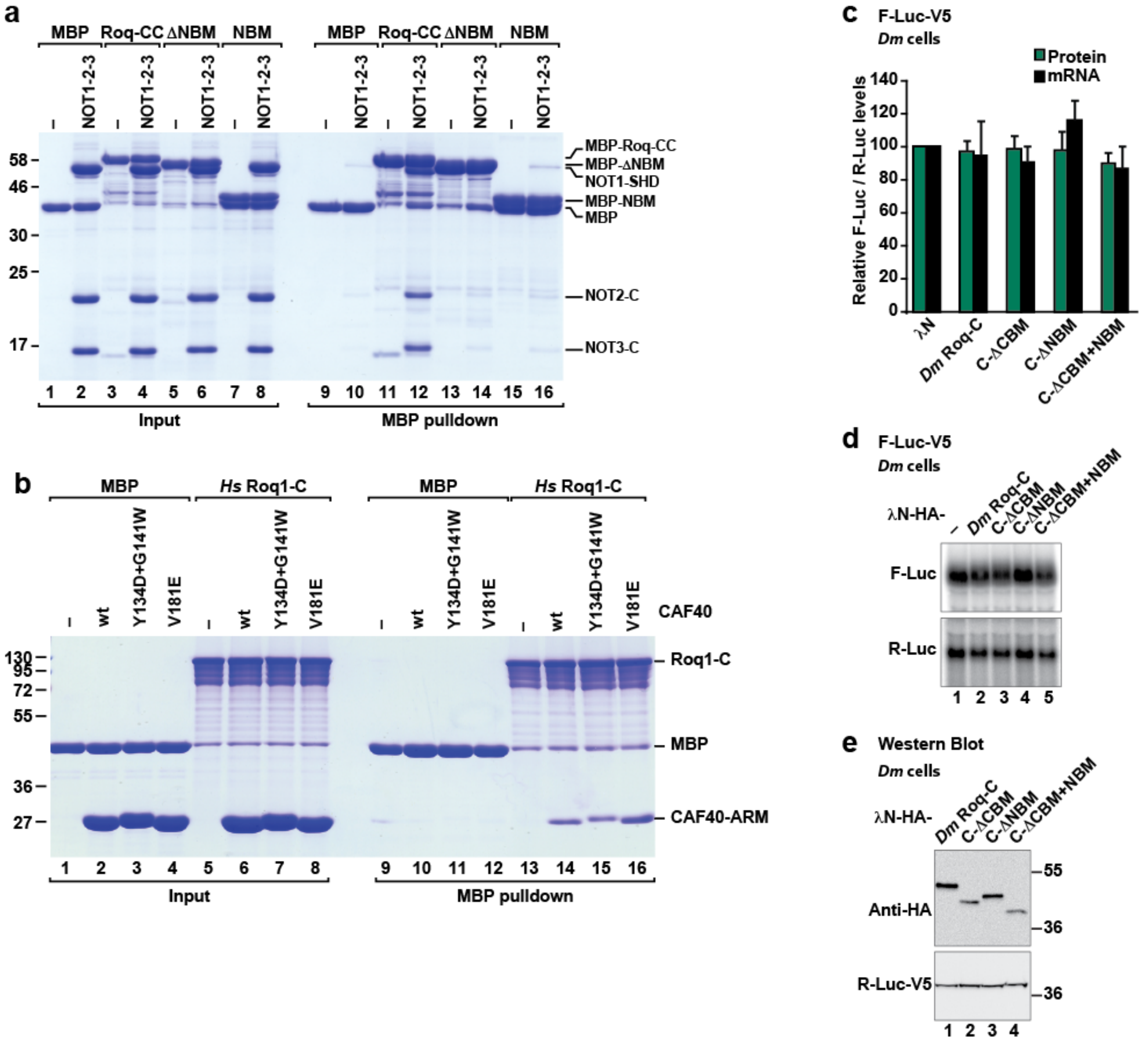

Supplementary Figure 5 | The CBM contributes to the activity of *Dm* Roquin. (**a**) *In vitro* MBP pulldown assay showing the interaction of MBP-tagged *Dm* Roq-CC, Roq-CC-ΔNBM or the NBM alone with the assembled NOT module (NOT1-2-3). MBP served as a negative control. (**b**) MPB pulldown assay showing the interaction of MPB-tagged *Hs* Roq1-C with the purified *Hs* CAF40-ARM domain (wild-type and the indicated mutants). MBP served as a negative control. (**c,d**) A tethering assay using the F-Luc-V5 reporter lacking λN binding sites and the indicated λN-HA-tagged *Dm* Roquin fragments was performed in *Dm* S2 cells as described in Fig. 3a,b. The corresponding experiment using the F-Luc-5BoxB reporter is shown in Fig. 5d,e. Error

bars represent s.d. from three independent experiments. (**e**) Western blot showing the equivalent expression of the λN-HA-tagged proteins used in panels (c,d) and in Fig. 5d,e. Protein size markers (kDa) are shown on the right of the panels.

Supplementary Figure 6

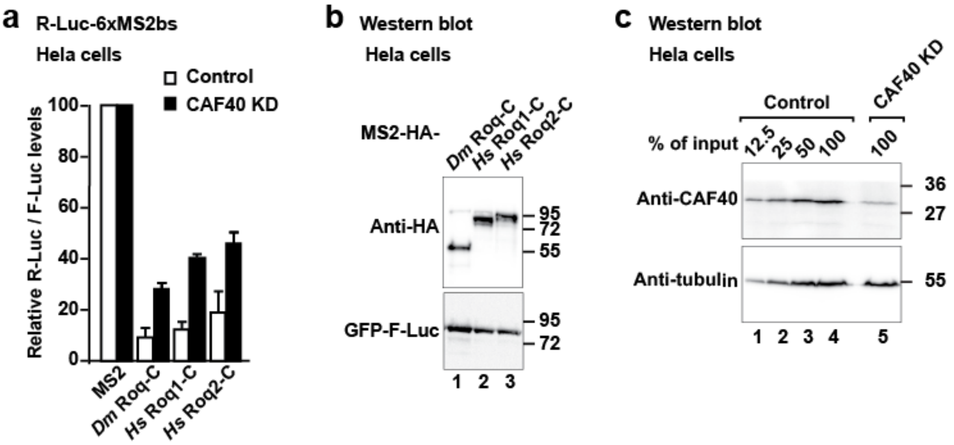

Supplementary Figure 6 | Depletion of CAF40 partially suppresses Roquin activity in HeLa cells. A tethering assay using the R-Luc-6xMS2bs was performed in HeLa cells depleted of CAF40 or treated with a control shRNA (Control). A plasmid expressing F-Luc served as a transfection control. For each condition, *Renilla* luciferase activity was measured, normalized to that of the F-Luc transfection control and set to 100% in cells expressing MS2-HA. (**a**) Mean values ± s.d. of the normalized R-Luc activities from three independent experiments. (**b**) Western blot analysis showing the equivalent expression of the MS2-HA-tagged proteins used in the tethering assay shown in panel (a). (**c**) Western blot showing the efficiency of the CAF40 knockdown. Dilutions of control cell lysates were loaded in lanes 1–4 to estimate the efficacy of the depletion. Tubulin served as a loading control.

Supplementary Figure 7

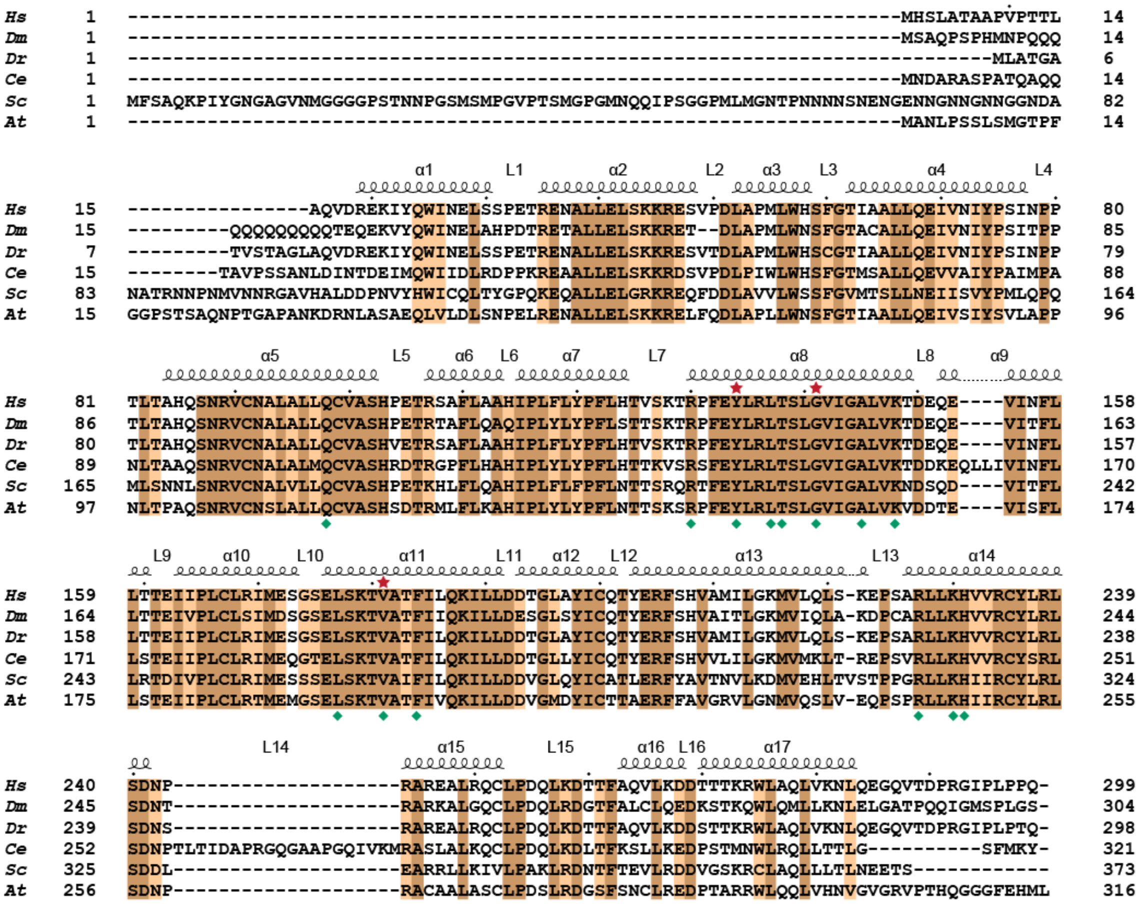

Supplementary Figure 7 | Structure-based sequence alignment of CAF40. Secondary structure elements as determined by the *Hs* CAF40-*Dm* Roquin structure are shown above the alignment. Residues conserved in all aligned species are highlighted by a brown background, and residues that show conservation of at least 70% are shown with an orange background. Conservation scores were calculated using the SCORECONS webserver[1]. Residues that directly contact the *Dm* Roquin CBM peptide are marked by green diamonds. The residues that were mutated in this study are indicated by red asterisks. Species abbreviations are as follows: *Hs*, *Homo sapiens*; *Dm*, *Drosophila melanogaster*; *Dr*, *Danio rerio*; *Ce*, *Caenorhabditis elegans*; *Sc*, *Saccharomyces cerevisiae*; *At*, *Arabidopsis thaliana*.

Supplementary Figure 8

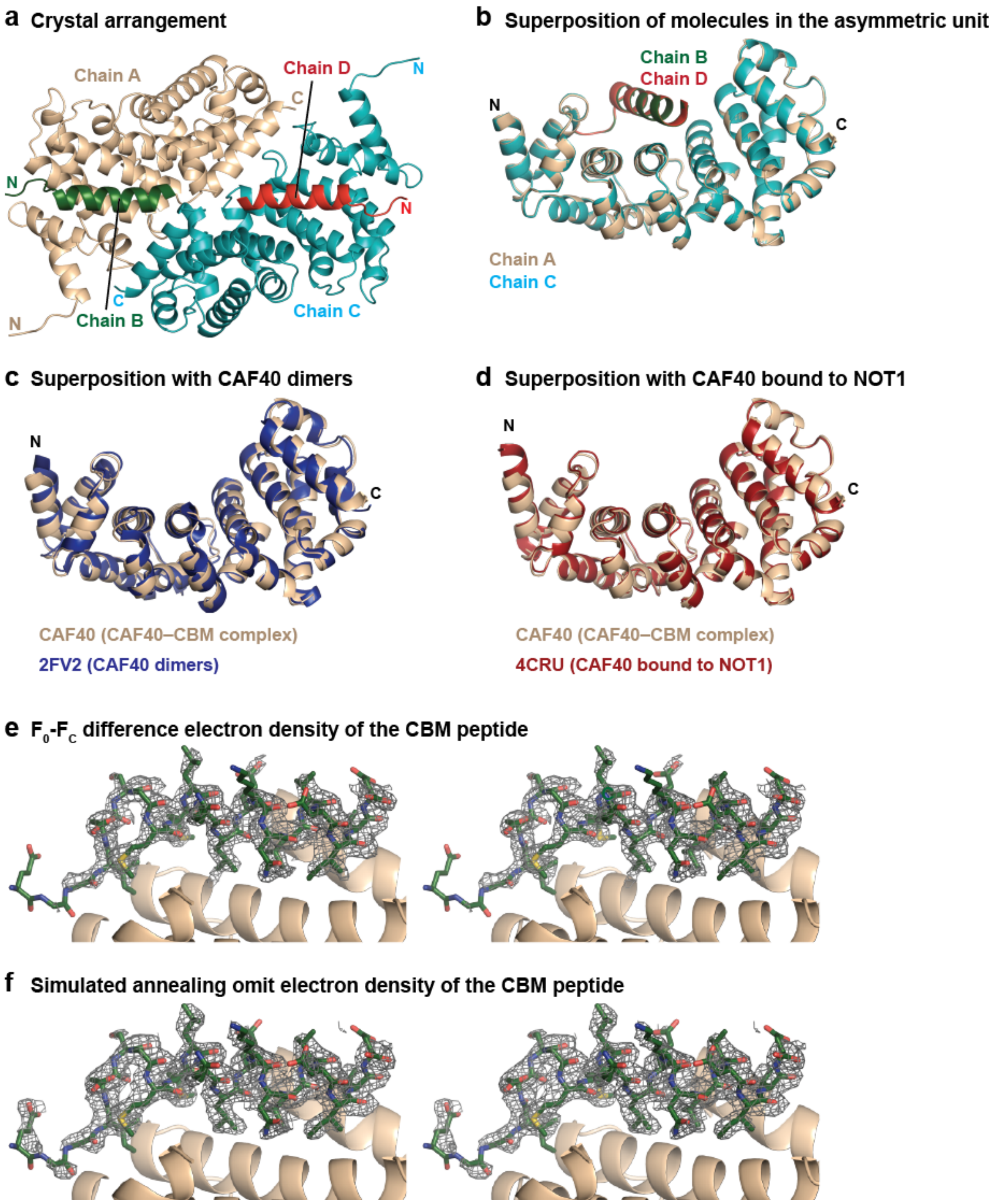

Supplementary Figure 8 | Structure of CAF40 bound to the *Dm* Roquin CBM peptide. (**a**) Crystal packing of the CAF40-Roquin CBM complex. CAF40 (chain A) and *Dm* Roquin CBM (chain B) from complex 1 are colored in light brown and green, respectively, whereas the molecules from complex 2 are colored in cyan (CAF40, chain

C) and red (CBM, chain D). (**b**) Superposition of the two CAF40-Roquin complexes from the asymmetric unit of the crystal. The colors are as described in (a). The structures superpose with an RMSD of 0.24 Å over 254 Cα atoms. (**c**) Superposition of the structure of the CAF40 ARM domain bound to the CBM (light brown, this study) with the structure of the isolated CAF40 ARM domain (blue, PDB 2FV2; ref. 37). (**d**) Superposition of the structure of the CAF40 ARM domain bound to the CBM (light brown, this study) with that of the CAF40 ARM domain bound to the NOT1 CN9BD (red, PDB 4CRU; ref. 2). (**e**) Stereo view showing the F_o-F_c difference electron density for the CBM peptide. The Roquin CBM peptide is shown in stick representation bound to CAF40 in cartoon representation. The difference electron density of the CBM peptide is shown as a grey mesh. The F_o-F_c-type map, which is contoured at 2.0 σ, was calculated using a refined CAF40 model before the CBM peptide was modelled. (**f**) Stereo view showing the $2F_o$-F_c simulated annealing composite omit map surrounding the CBM peptide. This $2F_o$-F_c map (which is largely devoid of model bias[2]), is shown around the CBM and contoured at 1.0 σ. It was generated with Phenix.Autobuild[3] using the final refined CAF40-Roquin model.

Supplementary Figure 9

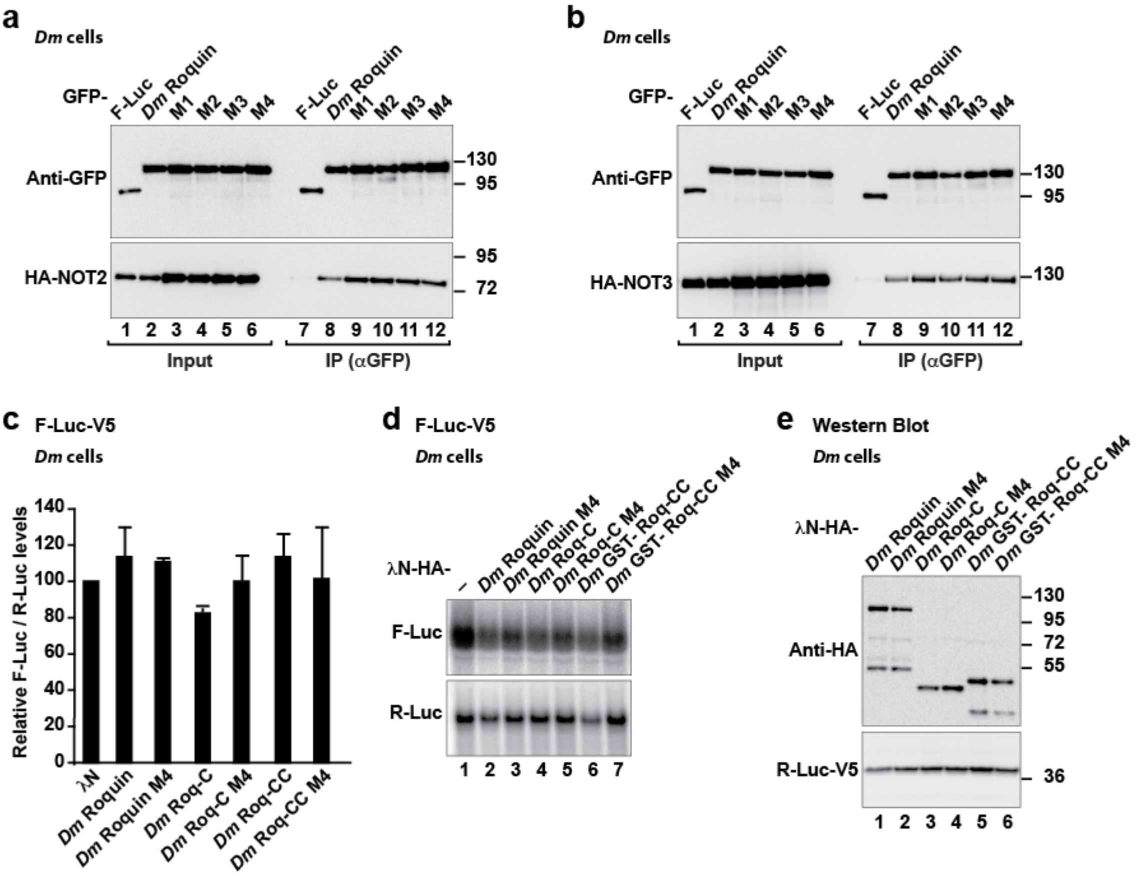

Supplementary Figure 9 | The CBM of *Dm* Roquin contributes to the recruitment of the CCR4-NOT complex. (**a,b**) Interaction of GFP-tagged *Dm* Roquin (wild-type and the M1, M2, M3 and M4 mutants; see Supplementary Table 1) with HA-tagged NOT2 and NOT3 in *Dm* S2 cells. F-Luc-GFP served as negative control. (**c,d**) A tethering assay using the F-Luc-V5 reporter lacking λN binding sites and the indicated λN-HA-tagged *Dm* Roquin mutants was performed in *Dm* S2 cells as described in Fig. 3a,b. The corresponding experiment using the F-Luc-5BoxB reporter is shown in Fig. 7f,g. Error bars represent s.d. from three independent experiments. (**e**) Western blot showing the equivalent expression of the λN-HA-tagged proteins used in panels (c,d) and Fig. 7f,g.

Supplementary Figure 10

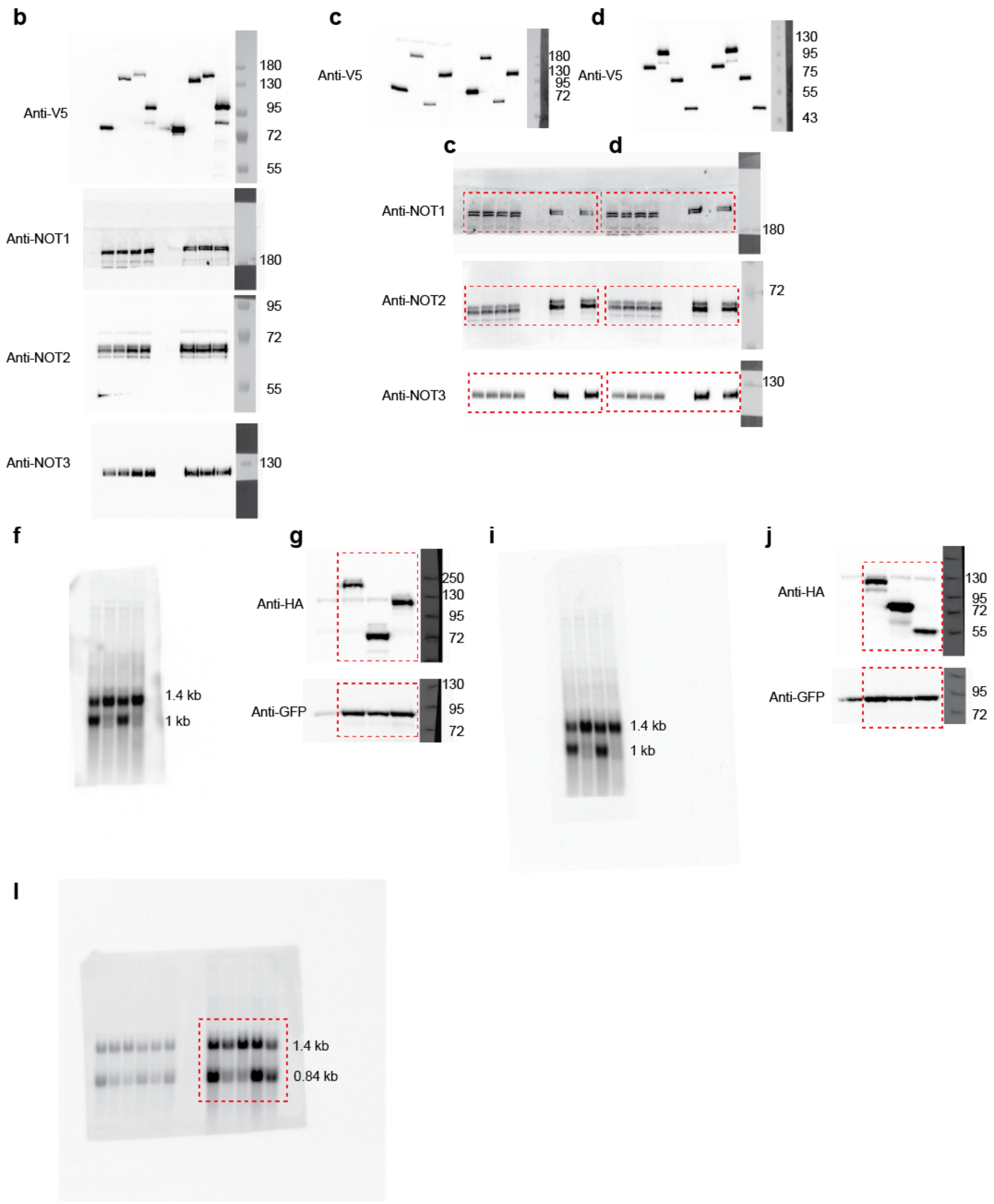

Supplementary Figure 10 | Original images of western and northern blots used in the corresponding panels in Fig. 1. The estimated sizes of the mRNA reporters without poly(A) and protein size markers (kDa) are shown on the right of the Northern and Western blots, respectively.

Supplementary Figure 11

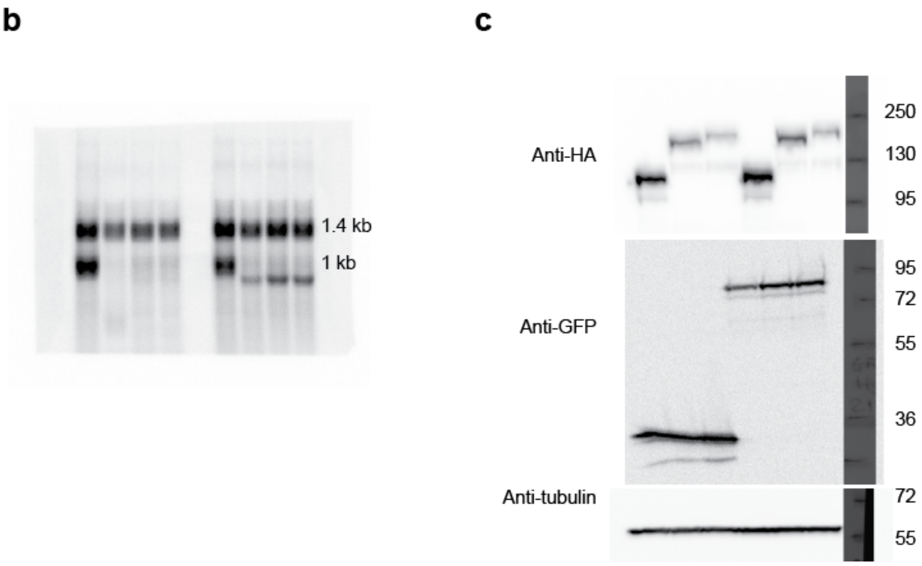

Supplementary Figure 11 | Original images of western and northern blots used in the corresponding panels in Fig. 2. The estimated sizes of the mRNA reporters without poly(A) and protein size markers (kDa) are shown on the right of the Northern and Western blots, respectively.

Supplementary Figure 12

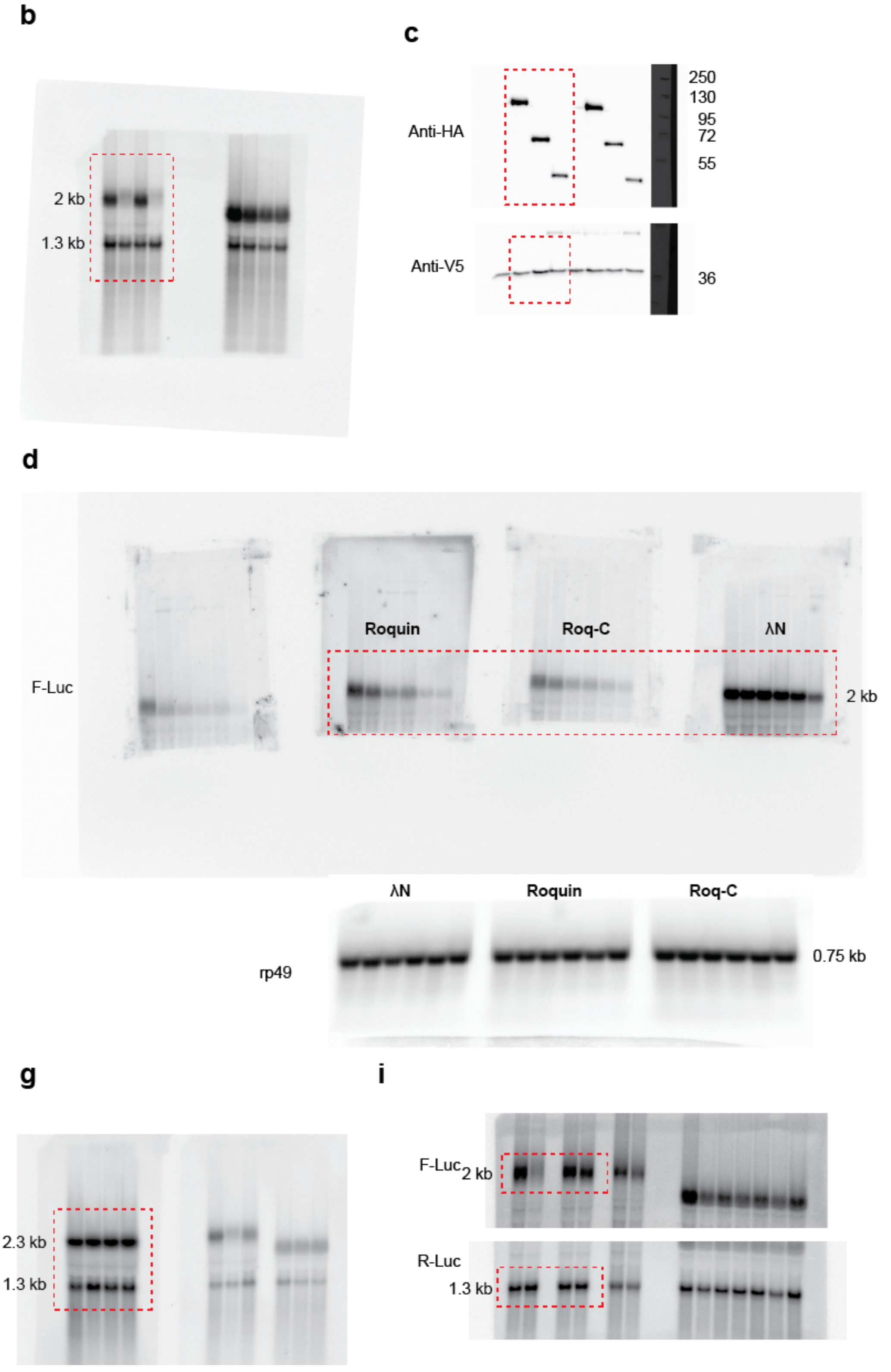

Supplementary Figure 12 | Original images of western and northern blots used in the corresponding panels in Fig. 3. The estimated sizes of the mRNA reporters without poly(A) and protein size markers (kDa) are indicated.

Supplementary Figure 13

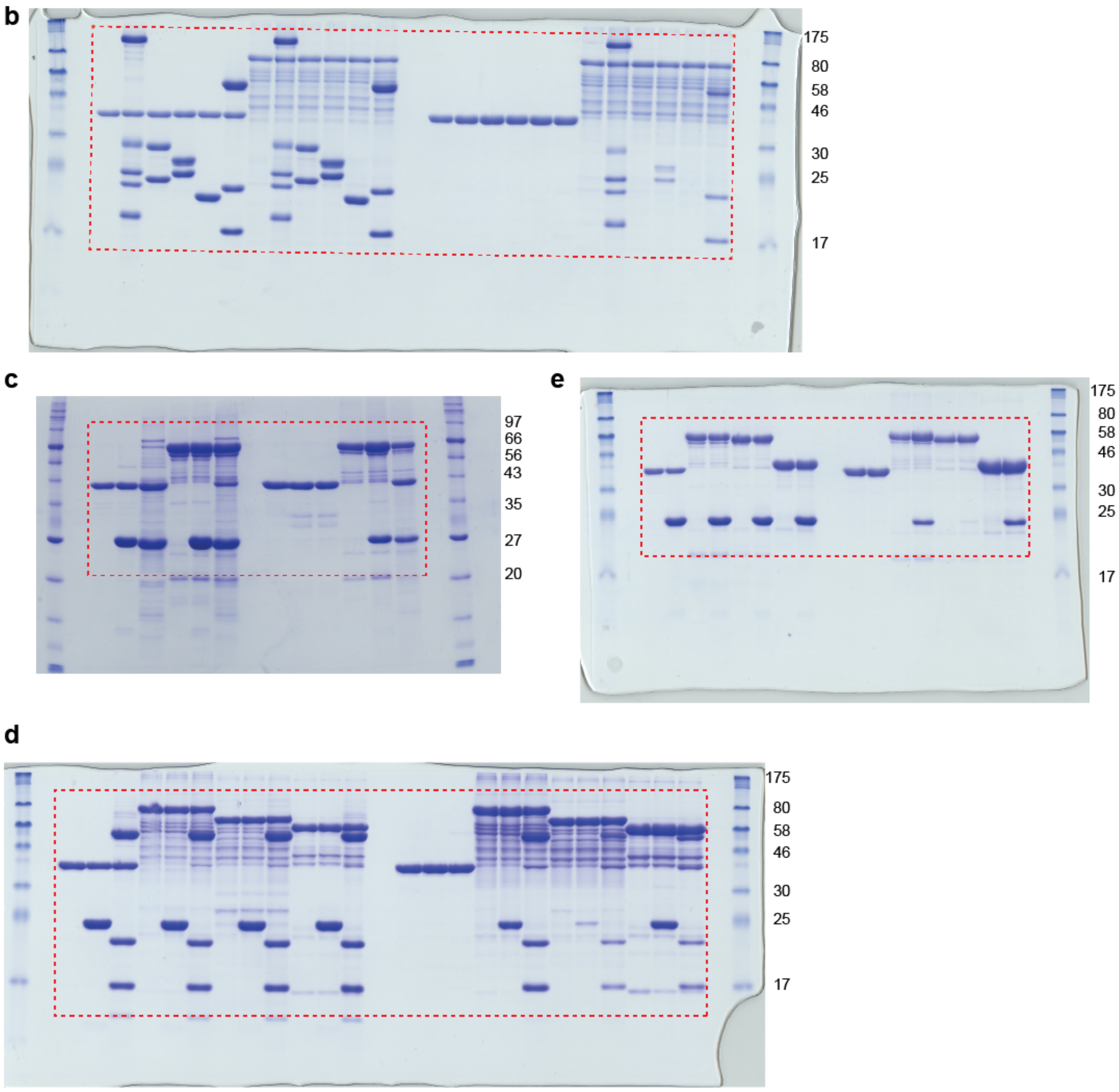

Supplementary Figure 13 | Original images of protein gels used in the corresponding panels in Fig. 4. Protein size markers (kDa) are shown on the right of the panels.

Supplementary Figure 14

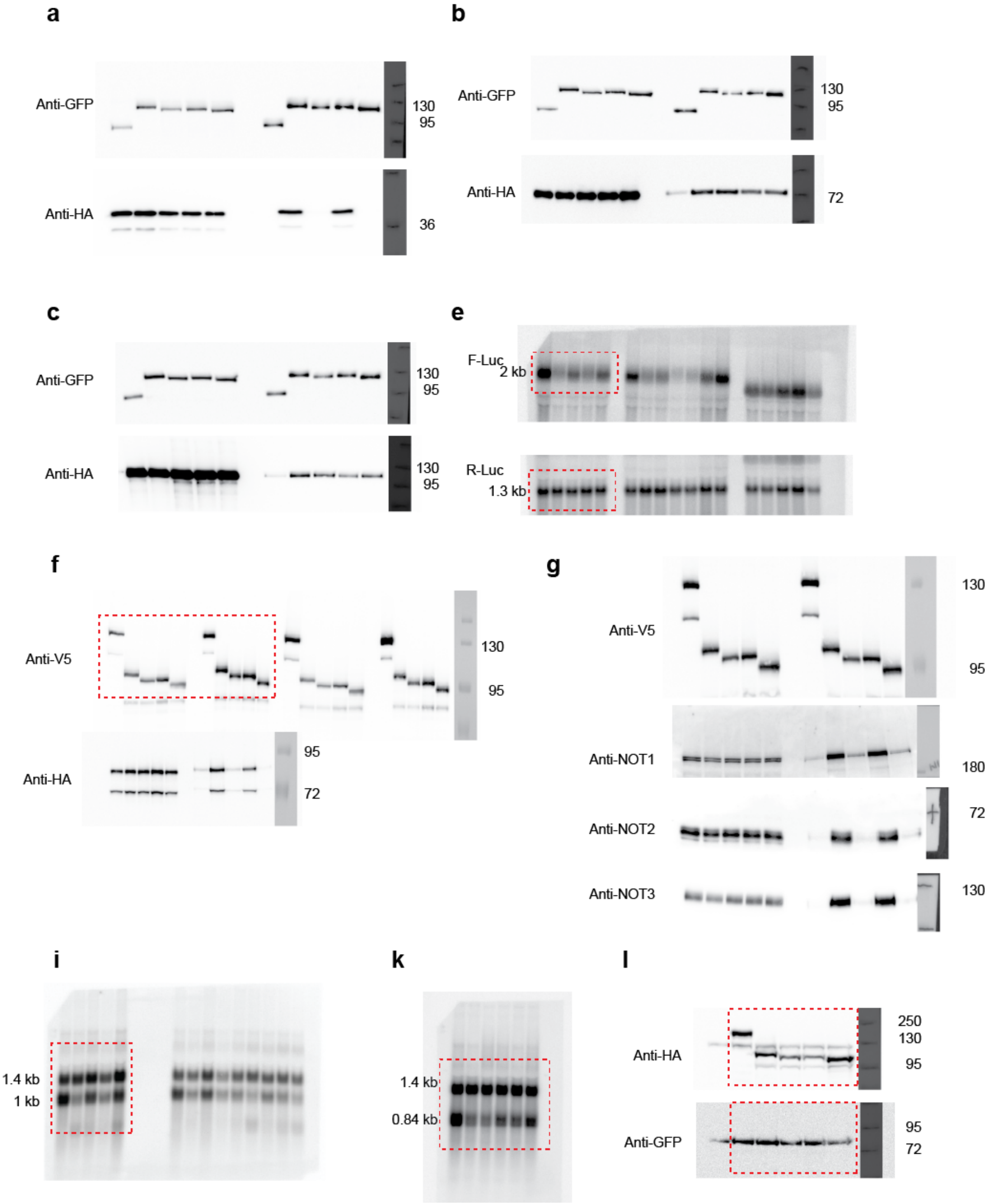

Supplementary Figure 14 | Original images of western and northern blots used in the corresponding panels in Fig. 5. The estimated sizes of the mRNA reporters without poly(A) and protein size markers (kDa) are indicated.

Supplementary Figure 15

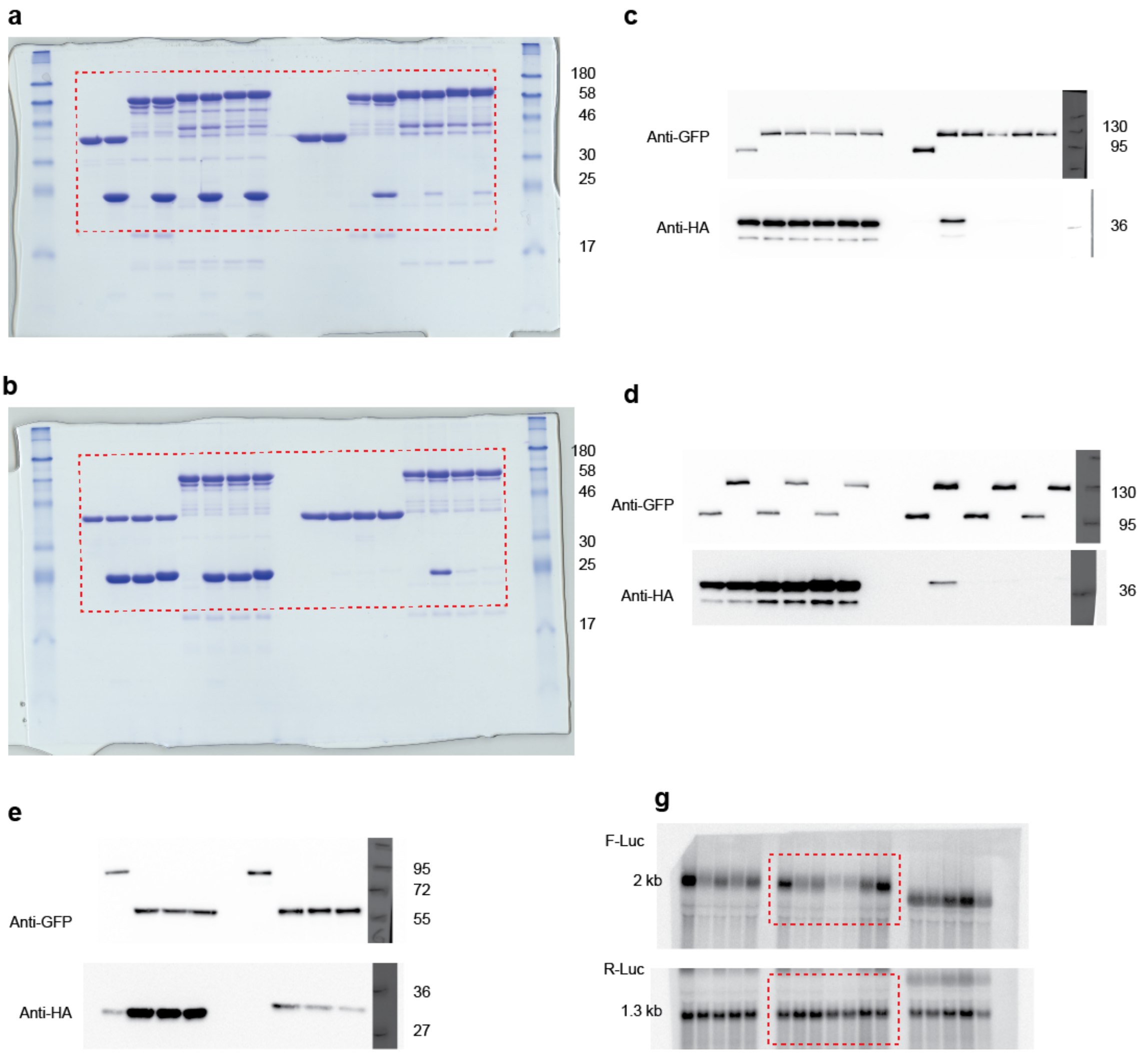

Supplementary Figure 15 | Original images of protein gels, western and northern blots used in the corresponding panels in Fig. 7. The estimated sizes of the mRNA reporters without poly(A) and protein size markers (kDa) are indicated

Supplementary Figure 16

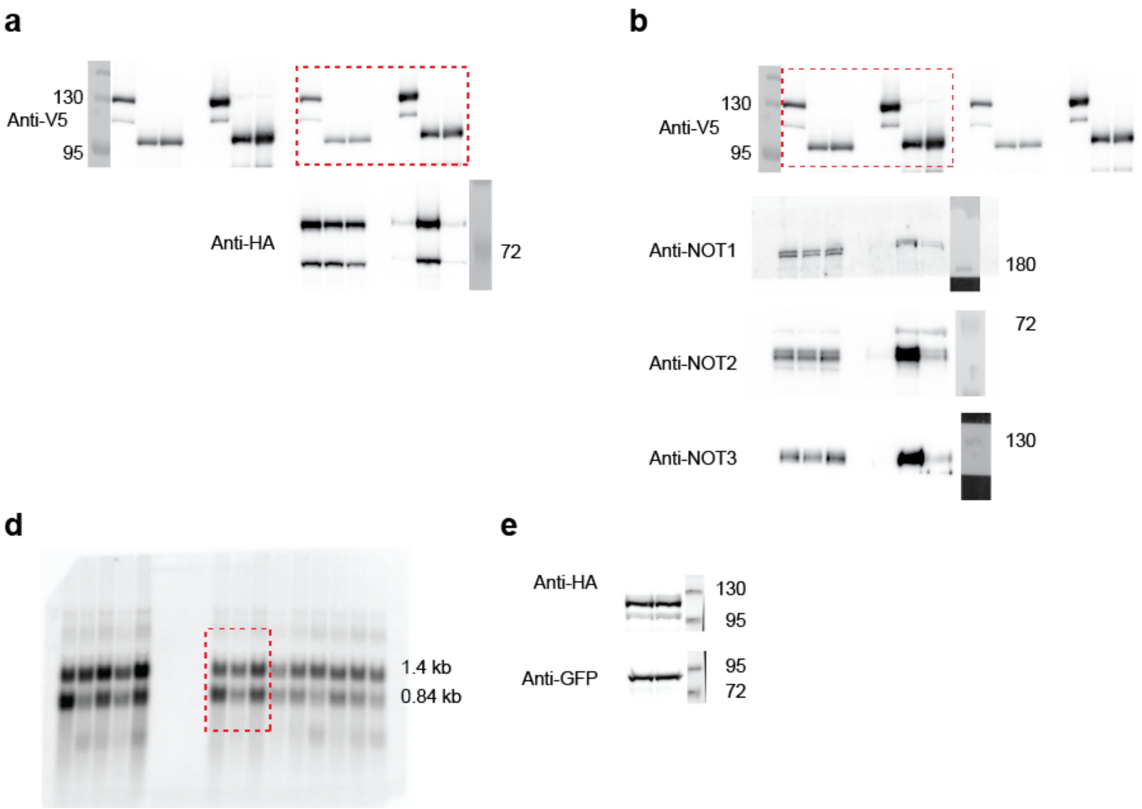

Supplementary Figure 16 | Original images of western and northern blots used in the corresponding panels in Fig. 8. The estimated sizes of the mRNA reporters without poly(A) and protein size markers (kDa) are indicated.

Supplementary Table 1. Constructs and mutants used in this study.

***Dm* Roquin (1-819)** (Uniprot Q9VV48)		**Comment**
Roquin	λN-HA-Roquin 1-819	
	GFP-Roquin 1-819	
	MS2-HA-Roquin 1-819	
	V5-SBP-Roquin 1-819	
Roq-N	λN-HA-Roquin 1-500	
	MS2-HA Roquin 1-500	
	V5-SBP-Roquin 1-500	
Roq-C	λN-HA-Roquin 501-819	
	MS2-HA-Roquin 501-819	
	V5-SBP-Roquin 501-819	
	MBP-Roquin 501-819-GB1-6xHis	
Roq-CN	λN-HA-GST-Roquin 501-702	
	λN-HA-GFP-Roquin 501-702	
	MBP-Roquin 501-702-GB1-6xHis	
Roq-CC	λN-HA-GST-Roquin 702-819	
	λN-HA-GFP-Roquin 702-819	
	MBP-Roquin 702-819-GB1-6xHis	
NBM	MBP-Roquin 725-755	NOT module-binding region
CBM	MBP-Roquin 790-812	CAF40-binding region
ΔNBM	MS2-HA-Roquin Δ725-755	NOT module-binding region
	GFP-Roquin Δ725-755	NOT module-binding region
	V5-SBP-Roquin Δ725-755	NOT module-binding region
Roq-C-ΔNBM	λN-HA-Roquin 501-819 Δ725-755	NOT module-binding region
Roq-CC-ΔNBM	MBP-Roquin 702-819 Δ725-755-GB1-6xHis	NOT module-binding region
ΔCBM	MS2-HA-Roquin Δ790-812	CAF40-binding region
	GFP-Roquin Δ790-812	CAF40-binding region
	V5-SBP-Roquin Δ790-812	CAF40-binding region
Roq-C-ΔCBM	λN-HA-Roquin 501-819 Δ790-812	CAF40-binding region
Roq-CC-ΔCBM	MBP-Roquin 702-819 Δ790-812-GB1-6xHis	CAF40-binding region
ΔCBM+NBM	MS2-HA-Roquin Δ725-755-Δ790-812	CAF40-binding region, NOT module-binding region
	GFP-Roquin Δ725-755-Δ790-812	CAF40-binding region, NOT module-binding region
	V5-SBP-Roquin Δ725-755-Δ790-812	CAF40-binding region, NOT module-binding region
Roq-C-ΔCBM+NBM	λN-HA-Roquin 702-819 Δ725-755-Δ790-812	CAF40-binding region, NOT module-binding region
Roq-CC ΔCBM+NBM	MBP-Roquin 702-819 Δ725-755-Δ790-812-GB1-6xHis	CAF40-binding region, NOT module-binding region
M1	GFP-Roquin I793E-L801E	CAF40-binding region
M2	GFP-Roquin L805E-V809E	CAF40-binding region
Roq-CC M2	MBP-Roquin 702-819 L805E-V809E-GB1-6xHis	CAF40-binding region
M3	GFP-Roquin L801E-L805E	CAF40-binding region

M4	GFP-Roquin I793E-L801E-L805E-V809E	CAF40-binding region
	λN-HA-Roquin I793E-L801E-L805E-V809E	CAF40-binding region
	MS2-HA-Roquin I793E-L801E-L805E-V809E	CAF40-binding region
	V5-SBP-Roquin I793E-L801E-L805E-V809E	CAF40-binding region
Roq-C M4	λN-HA-Roquin 501-819 I793E-L801E-L805E-V809E	CAF40-binding region
Roq-CC M4	λN-HA-GST-Roquin 702-819 I793E-L801E-L805E-V809E	CAF40-binding region
	MBP-Roquin 702-819 I793E-L801E-L805E-V809E-GB1-6xHis	CAF40-binding region
***Hs* Roquin1 (1-1133)** (Uniprot Q5TC82)		
Roquin1	MS2-HA-Roquin1 1-1133	
	V5-SBP-Roquin1 1-1133	
Roq1-N	MS2-HA-Roquin1 1-500	
	V5-SBP-Roquin1 1-500	
Roq1-C	MS2-HA-Roquin1 501-1133	
	V5-SBP-Roquin1 501-1133	
	MBP-Roq1 501-1133	
***Hs* Roquin2 (1-1191)** (Uniprot Q9HBD1)		
Roquin2	MS2-HA-Roquin2 1-1191	
	V5-SBP-Roquin2 1-1191	
Roq2-N	MS2-HA-Roquin2 1-500	
	V5-SBP-Roquin2 1-500	
Roq2-C	MS2-HA-Roquin2 501-1191	
	V5-SBP-Roquin2 501-1191	
***Hs* CNOT1** (Uniprot A5YKK6)		
CNOT1-MID+C	MBP-Hs CNOT1 1093-2371	Includes MIF4G, CN9BD, CD and SHD
CNOT1-MIF4G	6xHis-Hs CNOT1 1093-1317	MIF4G-like domain
CNOT1-CN9BD	MBP-Hs CNOT1 1351-1588	CNOT9-binding domain
CNOT1-CD	MBP-Hs CNOT1 1607-1815	Connector domain
CNOT1-SHD	MBP-Hs CNOT1 1833-2361	Superfamily homology domain
***Hs* CNOT2** (Uniprot Q9NZN8)		
CNOT2-C	MBP-Hs CNOT2 350-540	
***Hs* CNOT3** (Uniprot O75175)		
CNOT3-C	6xHis-Hs CNOT3 607-748	
***Hs* CNOT7** (Uniprot Q9UIV1)		
CNOT7	MBP-Hs CNOT7	

***Hs* CNOT9** (Uniprot Q92600)		
CNOT9-ARM	6xHis-HsCNOT9 19-285	
CNOT9-ARM V181E	6xHis-HsCNOT9 19-285-V181E	Disrupts Roquin binding
CNOT9-ARM 2xMut	6xHis-HsCNOT9 19-285-Y134D-G141W	Disrupts Roquin binding
***Dm* NOT9 (1-304)** (Uniprot Q7JVP2)		
CAF40	λN-HA-CAF40 1-304	
	GFP-CAF40 1-304	
CAF40 V186E	GFP-CAF40 V186E	Roquin-binding region
CAF40 2xMut	GFP-CAF40 Y139D-G146E	Roquin-binding region
CAF40-ARM	MBP-CAF40 25-291	Roquin-binding region
***Dm* NOT1** (Uniprot A8DY81)		
NOT1	λN-HA-NOT1	
CN9BD	λN-HA-CN9BD 1468-1717	CAF40-binding domain

Supplementary Table 2. Antibodies used in this study.

Antibody	Source	Catalog Number	Dilution	Monoclonal/ Polyclonal
Anti-HA-HRP	Roche	12 013 819 001	1:5,000	Monoclonal
Anti-GFP (for western blotting)	Roche	11 814 460 001	1:2,000	Monoclonal
Anti-GFP (for immunoprecipitations)	In house			Rabbit Polyclonal
Anti-*Dm* NOT1	Kind gift from E. Wahle	T6199	1:1,000	Rabbit Polyclonal
Anti-tubulin	Sigma-Aldrich	T6199	1:10,000	Monoclonal
Anti-V5	AbD Serotec	MCA1360GA	1:5,000	Monoclonal
Anti-mouse-HRP	GE Healthcare	NA931V	1:10,000	Monoclonal
Anti-*Hs* NOT1	In house		1:2,000	Rabbit polyclonal
Anti-*Hs* NOT2	Bethyl	A302-562A	1:2,000	Rabbit Polyclonal
Anti-*Hs* NOT3	Abcam	Ab55681	1:2,000	Monoclonal
Anti-*Hs* CAF40 (RQCD1)	Proteintech	22503-1-AP	1:1,000	Rabbit Polyclonal

Supplementary References

1. Valdar, W. S. J. Scoring residue conservation. *Proteins Struct. Funct. Bioinforma.* **48,** 227–241 (2002).

2. Terwilliger T. C. *et al.* Iterative-build OMIT maps: map improvement by iterative model building and refinement without model bias. *Acta Crystallogr. D Biol. Crystallogr.* **64,** 515–524 (2008).

3. Afonine, P. V. *et al.* Towards automated crystallographic structure refinement with phenix.refine. *Acta Crystallogr. D Biol. Crystallogr.* **68,** 352–367 (2012).
